# Pan-cancer analysis of RNA 5-methylcytosine reader (ALYREF)

**DOI:** 10.32604/or.2024.045050

**Published:** 2024-02-06

**Authors:** XING YE, ZHOUTING TUO, KAI CHEN, RUICHENG WU, JIE WANG, QINGXIN YU, LUXIA YE, AKIRA MIYAMOTO, KOO HAN YOO, CHI ZHANG, WURAN WEI, DENGXIONG LI, DECHAO FENG

**Affiliations:** 1 Samuel Oschin Comprehensive Cancer Institute, Department of Medicine, Department of Biomedical Sciences, Cedars-Sinai Medical Center, Los Angeles, CA, 90048, USA; 2 Department of Urology, The Second Affiliated Hospital of Anhui Medical University, Hefei, 230601, China; 3 Department of Urology, Institute of Urology, West China Hospital, Sichuan University, Chengdu, 610041, China; 4 Department of Pathology, Ningbo Diagnostic Pathology Center, Ningbo, 315021, China; 5 Department of Public Research Platform, Taizhou Hospital of Zhejiang Province Affiliated to Wenzhou Medical University, Linhai, 317000, China; 6 Department of Rehabilitation, West Kyushu University, Kanzaki-shi, 842-8585, Japan; 7 Department of Urology, Kyung Hee University, Seoul, 446 701, South Korea; 8 Department of Rehabilitation, The Affiliated Hospital of Southwest Medical University, Luzhou, 646000, China

**Keywords:** Pan-cancer, RNA 5-methylcytosine, ALYREF, Immuno-oncological effects

## Abstract

The increasing interest in RNA modifications has significantly advanced epigenomic and epitranscriptomic technologies. This study focuses on the immuno-oncological impact of ALYREF in human cancer through a pan-cancer analysis, enhancing understanding of this gene’s role in cancer. We observed differential ALYREF expression between tumor and normal samples, correlating strongly with prognosis in various cancers, particularly kidney renal papillary cell carcinoma (KIRP) and liver hepatocellular carcinoma (LIHC). ALYREF showed a negative correlation with most tumor-infiltrating cells in lung squamous cell carcinoma (LUSC) and lymphoid neoplasm diffuse large B-cell lymphoma (DLBC), while positive correlations were noted in LIHC, kidney chromophobe (KICH), mesothelioma (MESO), KIRP, pheochromocytoma and paraganglioma (PARD), and glioma (GBMLGG). Additionally, ALYREF expression was closely associated with tumor heterogeneity, stemness indices, and a high mutation rate in TP53 across these cancers. In conclusion, ALYREF may serve as an oncogenic biomarker in numerous cancers, meriting further research attention.

## Introduction

The core tenet of the central dogma in molecular biology, a landmark achievement of the 20th century, posits that genetic information can be exchanged between nucleic acids and proteins, two distinct classes of biological macromolecules [1]. This concept encompasses transcription and translation processes, where genetic information transfers from DNA to RNA and from RNA to proteins, and includes DNA replication. Additionally, RNA self-replication in some viruses (e.g., Tobacco Mosaic Virus) and reverse transcription of RNA into DNA in others (like certain oncogenic viruses) complement this rule [1–3]. In these processes, epigenomics plays a pivotal role in various cellular physiology processes in eukaryotes. It involves a biodiverse assembly of covalent modifications to histone proteins and nucleic acids, changing nucleosomes’ spatiotemporal arrangement, regulating chromatin’s three-dimensional conformation and nuclear topology, RNA splicing mechanisms, RNA binding protein location and activity, and transcribed elements of the non-protein coding genome. These elements work in concert to dynamically regulate chromatin structure and fine-tune gene expression, influencing biological properties [4]. Consequently, epigenome disruption can lead to the onset and progression of cancer through disordered transcriptional programs [5,6].

The growing understanding of DNA and RNA modifications’ biological functions has spurred advancements in epigenomic and epitranscriptomic technologies, revealing more than 17 and 160 different types of chemical modifications in DNA and RNA, respectively [7]. Epigenetic modifications involve the attachment, removal, and recognition of several chemical groups through specialized enzymes known as epigenetic “writers”, “erasers”, and “readers” [4,8,9]. While modifications in histones and DNA have been extensively investigated, covalent RNA modifications have primarily focused on the 5’ cap modification and the poly (A) tail, even though over one hundred types of chemical modifications have been identified in cellular RNAs since 1960s [10], notably following the sequencing of the first biological RNA in 1965 [11]. In recent decades, internal RNA modifications have gained attention for their versatile roles in cell fate, given the increasing awareness of RNA’s direct functional impact on gene expression through various classes of non-coding RNAs, such as microRNA and long ncRNA [12]. Among these, N6-methyladenosine is notable for its significant effects on normal life activities and diseases [13], serving as a promising biomarker and therapeutic targets [14–16]. Its functions include accelerate pre-mRNA processing, mRNA stability, splicing, nuclear transport, and translational ability [17–19]. Other RNA modifications include 5-methoxycarbonylmethyluridine, N6-methyladenosine, 5-methylcytosine (m5C), 7-methylguanosine, 5-methoxycarbonylmethyl-2-thiouridine, pseudouridine, N1-methyladenosine, and others [12].

The roles of m5C readers (YTHDF2, ALYREF, and YBX1) and writers (DNMTs and NSUNs), implicated in cellular metabolism and motility, are thought to regulate gene expression at the post-transcriptional stage [20]. This study aims to elucidate the immuno-oncological effect of ALYREF in human cancer through a comprehensive pan-cancer analysis, thereby deepening our understanding of this gene’s role in cancer.

## Materials and Methods

### Differential and prognostic analysis

Consistent with our previous studies [21,22], we extracted ALYREF (ENSG00000183684) expression data from a standardized TCGA pan-cancer dataset obtained from the UCSC database [23]. We also evaluated metastatic samples from primary tumor, TCGA-Skin Cutaneous Melanoma, and cancer-derived peripheral blood from primary blood (TCGA-Acute Myeloid Leukemia). A high-quality TCGA prognostic dataset was acquired from prior investigations [24]. After excluding samples with expression levels of 0 and those with follow-up periods shorter than 30 days, we logarithmically transformed each expression value log2 (x+1). Consequently, we obtained expression data for 38 malignancies and corresponding survival information (overall, disease-specific, disease-free, and progression-free survival), excluding cancers with fewer than 10 samples. ALYREF’s clinical associations with pancreatic cancer were also evaluated. Using the Cox proportional hazards regression model and the log-rank test, we analyzed ALYREF’s prognostic significance. We compared differential expression between tumor and normal samples by screening samples from solid tissue normal, cancer-peripheral blood, and primary tumors, and eliminating those with fewer than three samples. Differential significance was determined using signed rank tests and unpaired Wilcoxon rank sum tests.

### Tumor stemness, heterogeneity, and mutation landscape

We investigated the correlation between ALYREF expression and tumor stemness using the Spearman analysis method with two stemness indexes: epigenetically regulated RNA expression-based (EREG-EXPss) and RNA expression-based (RNAss) [25]. In addition, neoantigen (NEO) [26], tumor purity [26], and tumor mutation burden (TMB) obtained from the Genomic Data Commons (https://portal.gdc.cancer.gov/) and proceeded by MuTect2 software and R package “maftools” [27], and microsatellite instability (MSI) [28] were used to assess the relationship between tumor heterogeneity and ALYREF expression. We combined the information on mutations and gene expression, then sorted the samples of synonymous mutations. The frequency of gene mutations in high and low ALYREF expression groups was compared using the chi-square test, based on ALYREF’s median expression in each analyzed malignancy.

### Physical interactions and tumor immune microenvironment (TME)

We employed GeneMANIA to investigate potential physical interaction genes with ALYREF [29]. Additionally, we analyzed the associations of ALYREF mRNA expression with 24 inhibitory, 36 stimulatory checkpoints [26], and 150 immunoregulatory genes (chemokine, receptor, MHC, immunoinhibitory, immunostimulatory). The TME was assessed [30] using the Timer algorithm and the R package “IOBR” [31].

### Biological function of ALYREF

Given ALYREF’s notable association with urinary tumors, we examined its biological function in renal and prostate cancer cell lines. The CAKI-2 cell line, sourced from the National Collection of Authenticated Cell Cultures, was cultivated in matched complete medium. Our previous studies detail the cell culture procedures, real-time quantitative polymerase chain reaction (RT-qPCR), and cell counting kit-8 (CCK8) tests [32–34]. The primer sequences used were as follows, with glyceraldehyde-3-phosphate dehydrogenase (GAPDH) serving as an internal control: GAPDH: 5′-CTGGGCTACACTGAGCACC-3′ (forward) and 5′-TCCAAGTGGTCGTTGAGGGCAATG-3′ (reverse); ALYREF: 5′-TATGATCGCTCTGGTCGCAG-3′ (forward) and 5′-AGAGGGACGCCGTTGTACT-3′ (reverse). Additionally, the sequences of small interfering RNA (siRNA) of ALYREF were as follows: ALYREF si-1 sense: 5′-CGUGGAGACAGGUGGGAAAdTdT-3′; ALYREF si-1 antisense: 5′-UUUCCCACCUGUCUCCACGdTdT-3′. ALYREF si-2 sense: 5′-GGAGUCUCAGACGCCGAUAUUdTdT-3′; ALYREF si-2 antisense: 5′-AAUAUCGGCGUCUGAGACUCCdTdT-3′; ALYREF si-3 sense: 5′-GAACUCUUUGCUGAAUUUGGAdTdT-3′; ALYREF si-3 antisense: 5′-UCCAAAUUCAGCAAAGAGUUCdTdT-3′. Control sense: 5′-UUCUCCGAACGUGUCACGUdTdT-3′; Control antisense: 5′-ACGUGACACGUUCGGAGAAdTdT-3′.

### Statistical analysis

All analyses were conducted using software R (version 3.6.3) and its relevant packages. Unpaired Wilcoxon rank sum and signed rank tests assessed pairwise differences, while the Kruskal test was used for multiple sample sets. Spearman analysis evaluated correlations among continuous variables that failed the Shapiro‒Wilk normality test. Statistical significance was defined as a two-sided *p* < 0.05, with significance levels indicated as follows: *p < 0.05; **p < 0.01; ***p < 0.001.

## Results

### Differential expression and prognosis analysis

**Figure 1:**
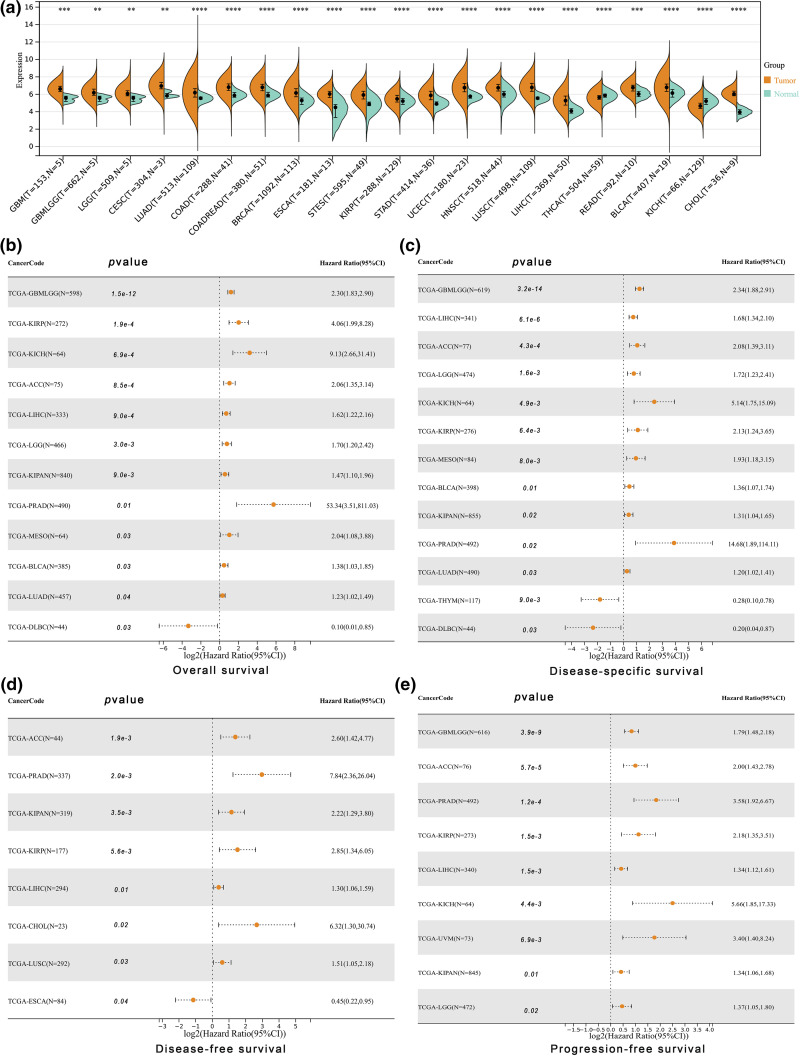
Differential expression and prognosis analyses at pan-cancer level. (a) ALYREF mRNA expression differences between tumor and normal samples with statistic significance at pan-cancer level; (b) forest plot showing the prognostic value of ALYREF for overall survival; (c) forest plot showing the prognostic value of ALYREF for disease-specific survival; (d) forest plot showing the prognostic value of ALYREF for disease-free survival; (e) forest plot showing the prognostic value of ALYREF for progression-free survival. **p < 0.01; ***p < 0.001; ****p < 0.0001.

**Figure 2:**
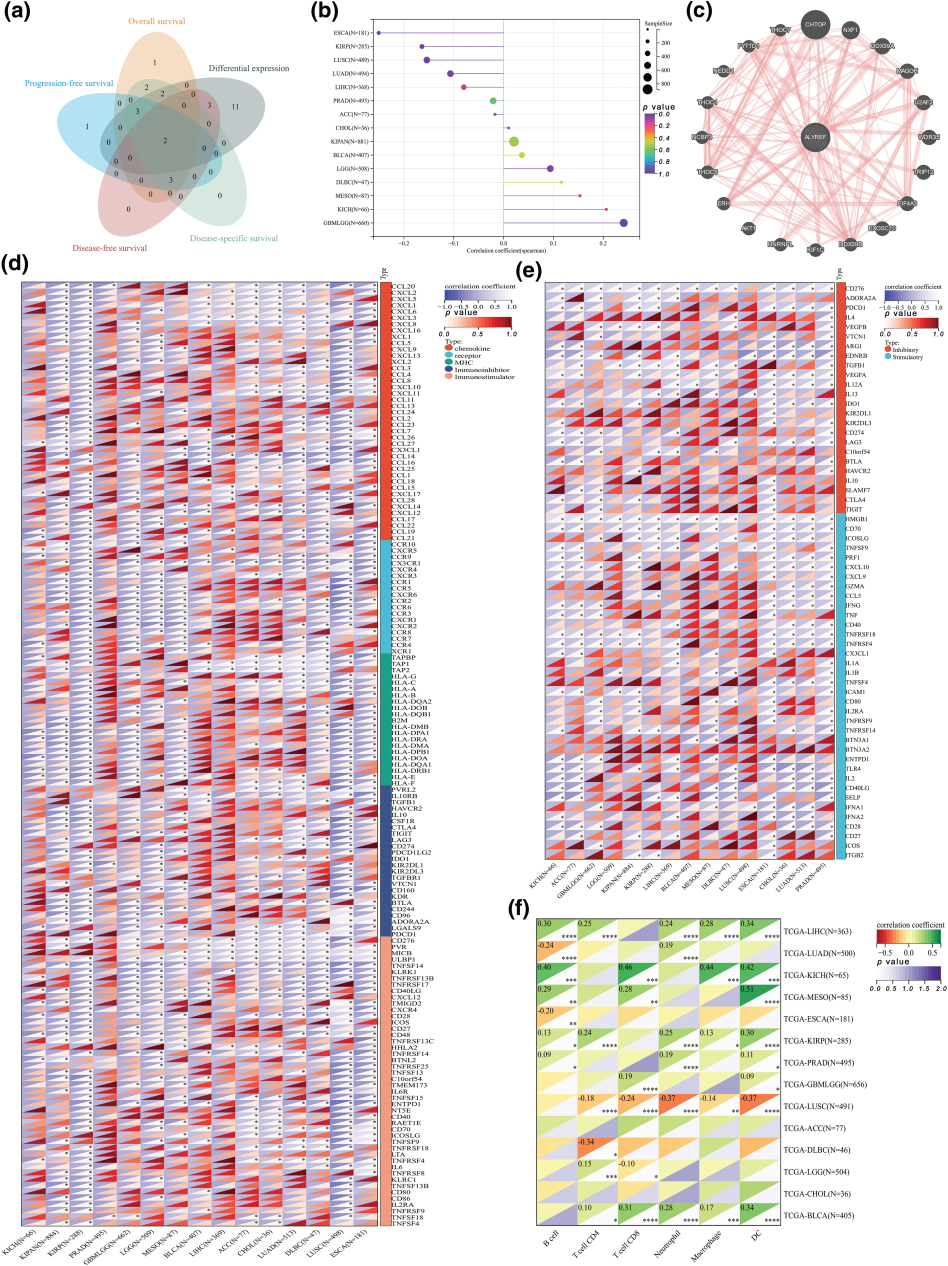
Clinical correlation and immune analyses. (a) Venn diagram showing the results of differential expression and various prognostic indexes; (b) the lollipop graph showing the correlation between ALYREF expression and age at pan-cancer level; (c) the physically interacted genes with ALYREF; (d) heat map showing the correlations between immunomodulatory genes and ALYREF at pan-cancer level; (e) heat map showing the correlations between immune checkpoints and ALYREF at pan-cancer level; (f) heat map showing the correlations between tumor-infiltrating cells and ALYREF at pan-cancer level. *p < 0.05; **p < 0.01; ***p < 0.001; ****p < 0.0001.

ALYREF exhibited significant differential expression in various human cancers compared to normal samples, with heightened expression in nineteen cancers and reduced expression in two (Fig. 1a). This gene was also closely associated with key prognostic indices including overall survival (Fig. 1b), disease-specific survival (Fig. 1c), disease-free survival (Fig. 1d), and progression-free survival (Fig. 1e) in numerous cancers. These included a majority of urinary tumors such as kidney chromophobe (KICH), pan-kidney cohort (KIPAN), kidney renal papillary cell carcinoma (KIRP), bladder urothelial carcinoma (BLCA), prostate adenocarcinoma (PRAD) and adrenocortical carcinoma (ACC)), glioma (GBMLGG), brain lower grade glioma (LGG), esophageal carcinoma (ESCA), lung squamous cell carcinoma (LUSC), cholangiocarcinoma (CHOL), mesothelioma (MESO), lymphoid neoplasm diffuse large B-cell lymphoma (DLBC), lung adenocarcinoma (LUAD), and liver hepatocellular carcinoma (LIHC). Notably, ALYREF’s relationship with KIRP and LIHC was statistically significant across all these prognostic indices (Fig. 2a). Furthermore, ALYREF mRNA expression showed significant age associations in six of these cancers (Fig. 2b), with both positive and negative correlations (Suppl. Fig. S1).

### Physical interactions and immune analysis

Twenty genes reported to interact physically with ALYREF were identified (Fig. 2c). ALYREF was positively correlated with most immunomodulatory genes in patients with urinary tumors (KICH, KIRP, KIPAN, PRAD, BLCA, and ACC), GBMLGG, LGG, MESO, and LIHC, showing particularly strong correlations with KICH and ACC (Fig. 2d). Similarly, ALYREF was positively associated with numerous immune checkpoints in these cancers, except for DLBC, LUSC, and ESCA (Fig. 2e). Notably, ALYREF demonstrated a significant correlation with CD276, an immunoglobulin superfamily member involved in regulating T-cell-mediated immune responses, in patients with urinary tumors (KICH, KIRP, KIPAN, PRAD, BLCA, and ACC), LIHC, GBMLGG, and LGG, especially in ACC, KIPAN, KIRP, PRAD, LIHC, GBMLGG, and LGG (r ≥ 0.5) (Fig. 2e). Consistent with these findings, ALYREF was negatively related to most tumor-infiltrating cells in LUSC and DLBC, while showing opposite correlations in LIHC, KICH, MESO, KIRP, PARD, and GBMLGG (Fig. 2f). Remarkably, KICH displayed notable correlations with B cells, CD8+ T cells, macrophages, and dendritic cells, and MESO exhibited a high correlation with dendritic cells (Fig. 2f).

### Tumor heterogeneity and stemness

**Figure 3:**
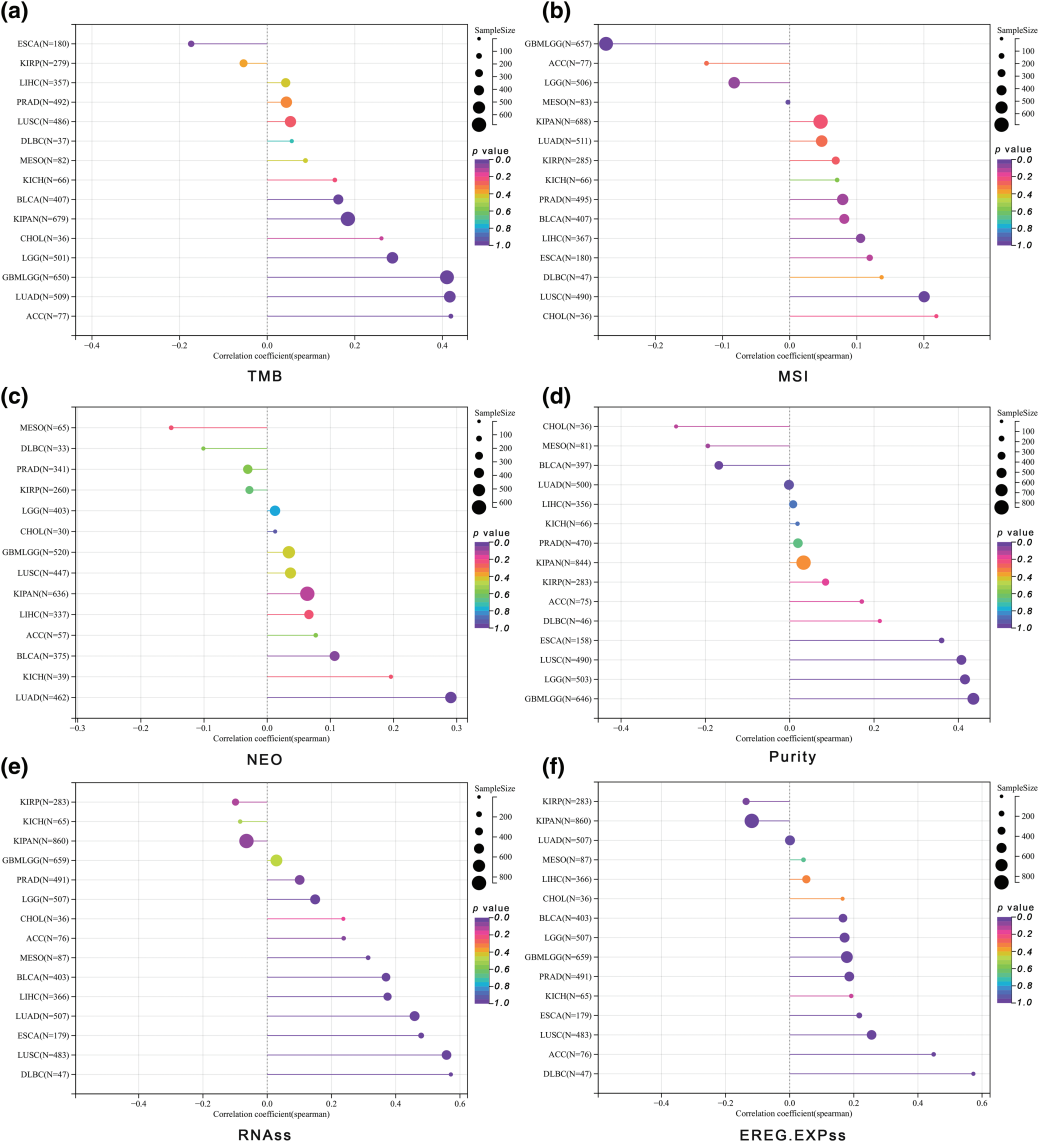
Tumor heterogeneity and stemness analyses. (a) The lollipop graph showing the correlation between ALYREF expression and TMB at pan-cancer level; (b) the lollipop graph showing the correlation between ALYREF expression and MSI at pan-cancer level; (c) the lollipop graph showing the correlation between ALYREF expression and NEO at pan-cancer level; (d) the lollipop graph showing the correlation between ALYREF expression and tumor purity at pan-cancer level; (e) the lollipop graph showing the correlation between ALYREF expression and RNAss at pan-cancer level; (f) the lollipop graph showing the correlation between ALYREF expression and EREG.EXPss at pan-cancer level.

We discovered significant correlations between ALYREF expression and various aspects of tumor heterogeneity and stemness. For TMB, ALYREF was significantly associated with GBMLGG (**r = 0.41**), LGG (r = 0.29), LUAD (**r = 0.41**), KIPAN (r = 0.18), BLCA (r = 0.16), ACC (**r = 0.42**) and ESCA (r = −0.17) (Fig. 3a). In terms of MSI, notable correlations were found with LUSC (r = 0.20), LIHC (r = 0.10), and GBMLGG (r = −0.27) (Fig. 3b). For NEO, ALYREF showed a significant relationship with LUAD (r = 0.26) (Fig. 3c). Regarding tumor purity, ALYREF was markedly associated with GBMLGG (**r = 0.44**), LGG (**r = 0.42**), ESCA (**r = 0.36**), LUSC (**r = 0.41**) and BLCA (r = −0.17) (Fig. 3d). In the context of tumor stemness, ALYREF was significantly correlated with LGG (r = 0.15), LUAD (**r = 0.49**), ESCA (**r = 0.48**), PRAD (r = 0.10), LUSC (**r = 0.56**), LIHC (**r = 0.37**), MESO (**r = 0.31**), BLCA (**r = 0.37**), ACC (r = 0.24), and DLBC (**r = 0.57**) for RNAss (Fig. 3e). For EREG.EXPss, significant correlations were noted with GBMLGG (r = 0.18), LGG (r = 0.17), ESCA (r = 0.22), PRAD (r = 0.19), LUSC (r = 0.26), BLCA (r = 0.17), ACC (**r = 0.45**), DLBC (**r = 0.57**), KIRP (r = −0.14), and KIPAN (r = −0.12) (Fig. 3f).

### Mutation analysis

**Figure 4:**
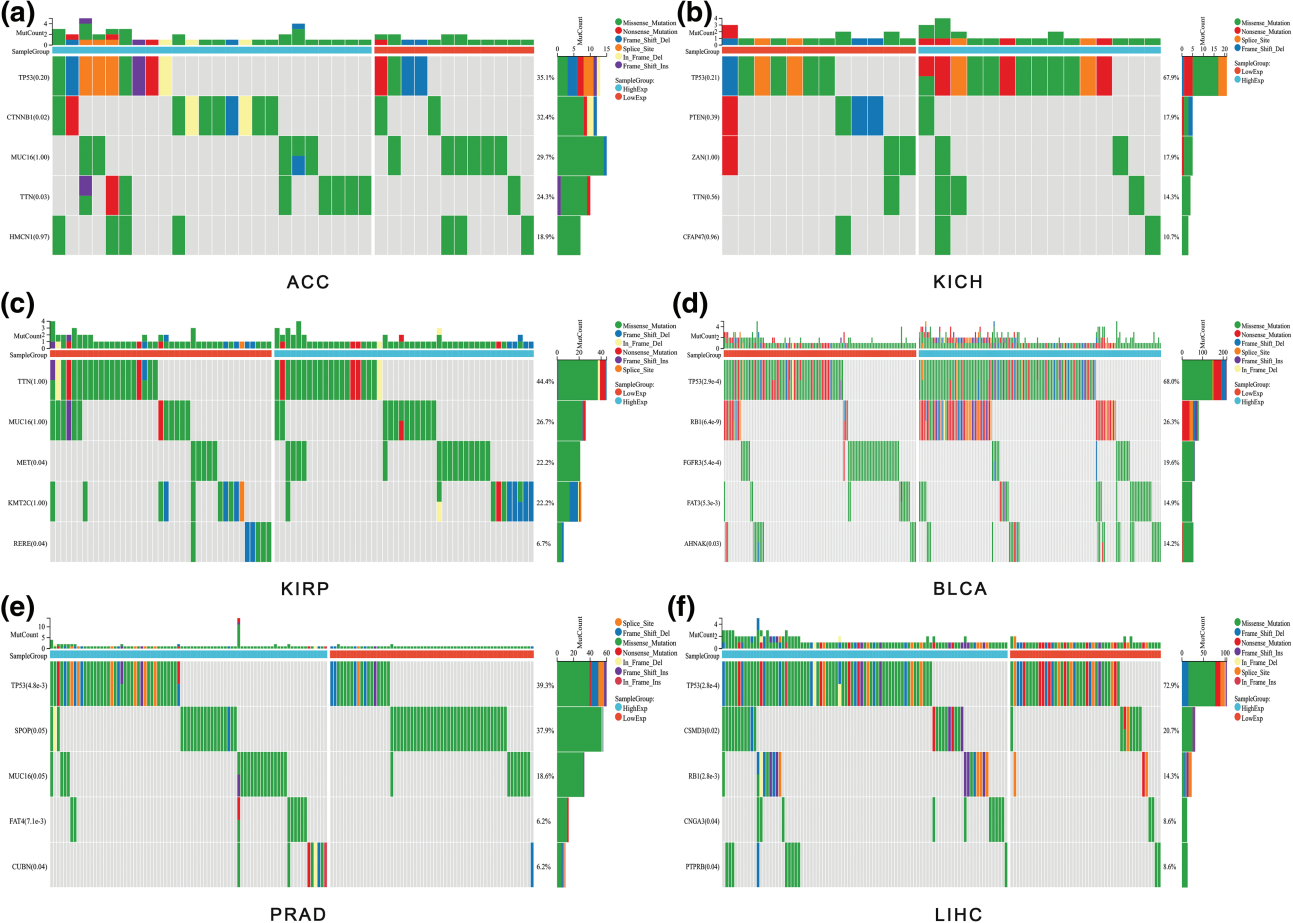
Mutation analysis. (a) Waterfall plot showing the gene mutation frequency differences between high-and low-ALYREF expression groups in ACC; (b) waterfall plot showing the gene mutation frequency differences between high-and low-ALYREF expression groups in KICH; (c) waterfall plot showing the gene mutation frequency differences between high- and low-ALYREF expression groups in KIRP; (d) waterfall plot showing the gene mutation frequency differences between high- and low-ALYREF expression groups in BLCA; (e) waterfall plot showing the gene mutation frequency differences between high-and low-ALYREF expression groups in PRAD; (f) waterfall plot showing the gene mutation frequency differences between high-and low-ALYREF expression groups in LIHC. Note: group was determined by the median value of ALYREF expression.

Given ALYREF’s prominent role in urinary tumors and LIHC, we analyzed their mutation landscapes (Fig. 4). TP53 emerged as the most mutated gene in these cancers (Figs. 4a and 4b, 4d–4f). Notable mutations included CTNNB and TTN in ACC (Fig. 4a), MET and RERE in KIRP (Fig. 4c), TP53, RB1, FGFR3, FAT3, and AHNAK in BLCA (Fig. 4d), TP53, FAT4, and CUBN in PRAD (Fig. 4e), TP53, CSMD3, RB1, CNGA3 and RTPRB in LIHC (Fig. 4f), showing significant variation between high- and low-expression ALYREF groups. These findings underscore the importance of TP53 in these cancers.

### Cell proliferation

**Figure 5:**
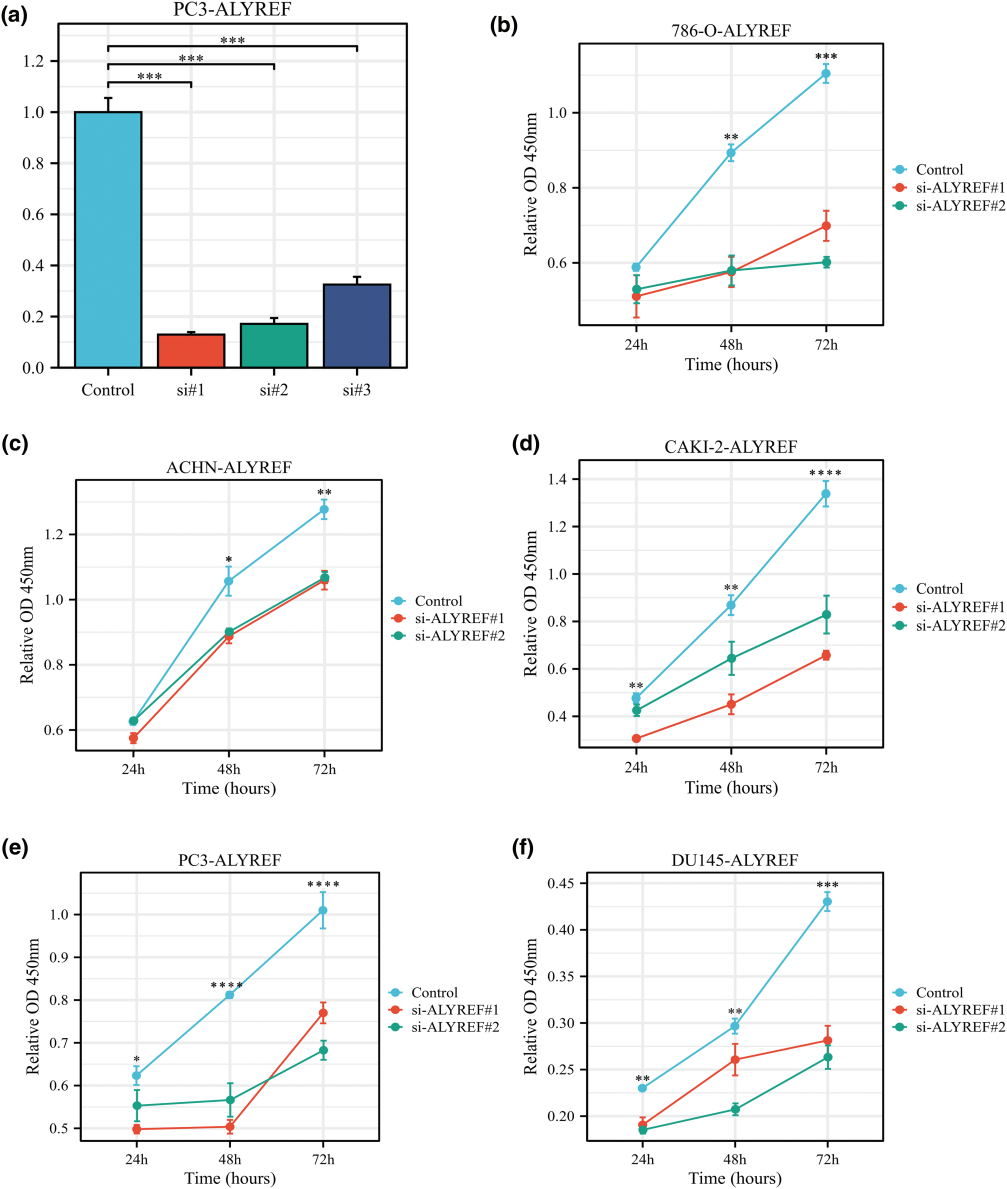
Effect of ALYREF on the biological behaviors of renal cancer and prostate cancer cell lines. (a) RT‒qPCR results of ALYREF siRNAs; (b) Effect of ALYREF siRNAs on 786-O cells using CCK8 assay; (c) Effect of ALYREF siRNAs on ACHN cells using CCK8 assay; (d) Effect of ALYREF siRNAs on CAKI-2 cells using CCK8 assay; (e) Effect of ALYREF siRNAs on PC3 cells using CCK8 assay; (f) Effect of ALYREF siRNAs on DU145 cells using CCK8 assay. *p < 0.05; **p < 0.01; ***p < 0.001; ****p < 0.0001.

Using the RT-qPCR assay, we observed that ALYREF siRNA1, siRNA2, and siRNA3 effectively reduced ALYREF mRNA expression in PC3 cells (Fig. 5a). Based on these results, siRNA1 and siRNA2 were selected for subsequent experiments. We found that these siRNAs significantly inhibited the proliferation of 786-O, ACHN, CAKI-2, PC3 and DU145 cells (Figs. 5b–5f).

## Discussion

The epigenome plays a crucial role in a wide array of cellular processes and is vital for the survival of living organisms. Recent studies have highlighted the use of epigenetic markers, like histone acetylation and methylation marks on cell-free nucleosomes, as proxies for RNA-based transcriptional profiling [35]. The development, progression, and metastasis of tumors are closely linked to aberrant epigenomes, which encompass DNA methylation, histone modification, nucleosome remodeling, and RNA level changes [36]. Over recent decades, significant progress has been made in understanding the functional importance of RNA modifications in regulating the processing and function of both coding and non-coding RNAs, thereby shaping diverse gene expression programs.

The identification of DNA m5C dates back to the 1950s, and it was later recognized that m5C modification, a common alteration in various RNA species, occurs when a methyl group from the donor molecule, typically S-adenosyl-methionine, attaches to thefifth carbon position of the cytosine base in RNA [37,38]. In recent years, it has become evident that m5C RNA modification plays a critical role in controlling how both coding and non-coding RNAs regulate RNA metabolism and function. There is increasing evidence that m5C influences RNA stability, translation, nuclear export, and cleavage, thereby affecting cell division, differentiation, apoptosis, stress responses, and other biological processes [6,39]. While m5C is found in a broad range of RNAs, it is most common in eukaryotic tRNAs and rRNAs and is typically detected using RNA bisulfite sequencing, which yields efficient and abundant results [40–43]. Other detection methods include RNA methyltransferases crosslinked to RNA targets and m5C antibody-based immunoprecipitation techniques [40]. However, methods for identifying m5C sites in RNAs other than tRNA and rRNA are less reliable, underscoring the urgent need for new computational methods and improved bisulfite sequencing techniques [44,45].

*In vitro* and *in vivo* studies have identifiedthe RNA methyltransferase NSUN2 as primarily enzyme catalyzing m5C production in mRNAs, with m5C specifically recognized by the mRNA export adaptor ALYREF [46]. Recent research has linked ALYREF to the development of various tumors, including bladder cancer [47,48], breast cancer [49], neuroblastoma [50], hepatocellular carcinoma [51,52], glioblastoma [53], and non-small cell lung cancer [54]. These findings underscore the gene’s critical role in human cancer. Beyond these experimentally validated cancers, our study observed differential ALYREF expression in most cancers and its prognostic potential in KICH, KIRP, PRAD, ACC, ESCA, CHOL, MESO, and DLBC, particularly in urinary tumors. Additionally, ALYREF inhibition significantly impaired renal and prostate cancer cell proliferation *in vitro*. Recent studies indicate that aging is accompanied by progressive epigenetic alterations in both dividing and nondividing cells [55], with epigenetic changes proposed as a hallmark of aging by 2023 [56]. Aging is a risk factor for various age-related diseases, including cancers [57–60] and degenerative diseases [61–64]. Our study found that ALYREF expression was significantly related to aging, confirming the link between epigenomics, aging, and cancer. We also identified several ALYREF interaction genes for future cancer research.

In this study, correlation analyses between ALYREF expression and immunomodulatory genes, immune checkpoints, and tumor-infiltrating cells showed consistent results. We observed a significant positive correlation between ALYREF and most tumor-infiltrating cells in urinary tumors, LIHC, GBMLGG and LGG. Notably, ALYREF also correlated positively with CD276. During immune response, T cell activation depends on two signals: the interaction between the T cell receptor and the peptide-major histocompatibility complex on antigen-presenting cells, and the engagement of CD28 receptor family members on T cells with B7 ligand family members on antigen-presenting cells [65–68]. This costimulatory signaling is crucial for both helper and cytotoxic T cells, enhancing their activation, proliferation, and differentiation, and leading to cytokine reception [65–67]. CD276, also known as B7-H3, is a type I transmembrane protein that belongs to the second signal pathway in immune responses [69–71]. Although broadly expressed in various tissues (such as liver and prostate) and cell types at the mRNA level, its protein expression is limited due to post-transcriptional regulation [66]. CD276 is believed to be involved in regulating the T-cell-mediated immune response and may play a protective role in tumor cells by inhibiting natural-killer mediated cell lysis [72]. Research indicates that despite the widespread mRNA expression of this gene in both normal tissues and solid tumors, the protein predominantly occurs in tumor tissues. This expression correlates with negative prognosis and poor clinical outcomes in patients [66,72]. Consequently, we hypothesize that ALYREF may increase CD276 protein levels in antigen-presenting cells, thereby restricting the function of CD8+ T cells. However, ALYREF mRNA expression shows a negative correlation with tumor-infiltrating cells in LUSC. Given that this gene is upregulated in most cancers, including LUSC, we suggest that ALYREF could contribute to the formation of immunodeficiency niches by reducing the number of tumor-infiltrating cells, thus exacerbating LUSC progression. Estimating tumor purity using high-throughput genomic and epigenomic data presents an alternative to cell sorting technologies, such as Fluorescent-Activated Cell Sorting or Magnetic-Activated Cell Sorting. This approach is advantageous for assessing the proportion of cancer cells in solid tumor samples due to the time-consuming and costly nature of experimental validation [73–75]. In addition to fewer immune cells, LUSC patients with high ALYREF expression tend to exhibit greater tumor purity, which suggests a propensity for worse outcomes. Although no significant correlation was found between ALRREF expression and tumor-infiltrating cells in patients with GBMLGG, LGG, and ESCA, patients with high expression of this gene often have higher tumor purity and are likely to experience poorer prognoses. NEO, produced by cancer cell mutations, possesses strong immunogenicity and is exclusively expressed in tumor cells, making it an attractive therapeutic target [76].

Moreover, TMB defined by the number of somatic mutations, including synonymous and intron mutations, per 38 Mb of genome area for targeted sequencing, has shown that higher TMB correlates with improved survival in bladder cancer patients receiving immune checkpoint inhibitor treatments [77–80]. LUAD, representing almost 40% of lung cancer cases, is a prevalent subtype and is noted for its high morbidity and mortality rates globally [37,81]. In China, lung cancer has been the most rapidly increasing malignancy over the past 30 years, predominantly comprising histological subtypes such as adenocarcinoma and squamous cell carcinoma, which make up 80%–85% of cases, with the remainder being small cell lung cancer (atezolizumab plus etoposide/platinum) [58,82]. In our study, a positive association was observed between ALYREF and both NEO and TMB in LUAD, suggesting that patients with high ALYREF expression might respond better to immunotherapy. Additionally, GBMLGGs, highly aggressive cancers originating in the neuroepithelial layer, and ACC, despite its low incidence, often lead to immediate metastases, precluding surgical interventions [83–85]. Patients with overexpressed ALYREF in these cancers might also benefit from immunotherapy. As cancer progresses, a differentiated phenotype is lost, and cancer cells acquire progenitor- and stem-cell-like characteristics, indicating that undifferentiated primary tumors are more prone to metastasis and associated with worse prognosis [25]. In Hodgkin’s lymphoma series, including DLBC, the incidence ranges from 30% to 58%, and the recommended radiotherapy dose post-chemotherapy is 30 to 46 Gy; yet, over 30% of DLBC patients relapse [86,87]. A clear positive correlation was found between ALYREF expression and both RNAss and EREG.EXPss in DLBC patients, hinting at a potentially poorer prognosis for those with higher ALYREF expression.

TP53, the most frequently mutated gene in human cancers, involves multiple pathways for tumor growth inhibition [88–91]. However, TP53 mutations often lead to p53 inactivation, enabling tumor cells to evade death and grow rapidly [89,92,93]. Originally identified as a key regulator in acute DNA damage response, recent studies have uncovered additional TP53 downstream targets and pathways [92–95], including those related to stemness, metabolism, redox biology, genomic integrity, and tumor suppression through non-cell autonomous signaling [88,90,92,95]. Consistently, our study found TP53 to be the most mutated gene in urinary tumors and LIHC, with a higher mutation frequency in patients expressing higher ALYREF, underscoring the significance of TP53 in cancer. Given the prevalence of TP53 mutations in cancers, efforts are ongoing to restore mutant p53 functionality, aiming to induce tumor cell death and eradication [89]. In this study, we observed that our research suggests that ALYREF has a broad potential role in various cancers, particularly in urinary tumors and LIHC.

## Conclusions

Our findings suggest that ALYREF could serve as an oncogenic biomarker in many cancer patients and should be given more attention by researchers.

## Supplementary Materials

**Figure S1:**
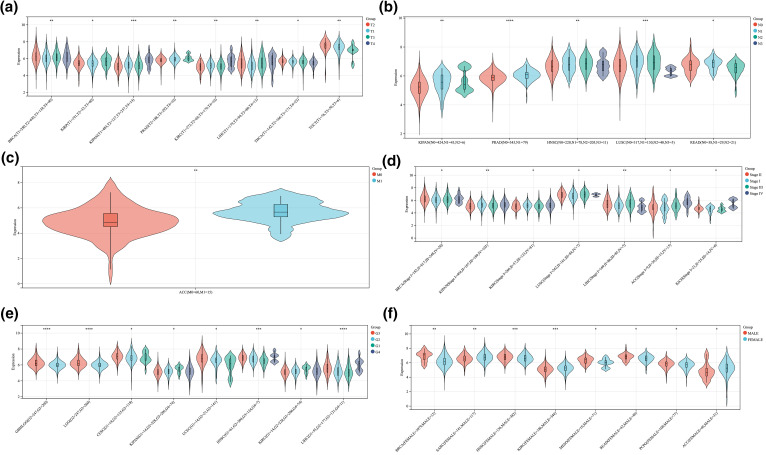
The correlation between ALYREF expression and clinical features at pan-cancer level. (a) ALYREF expression differences among different T stages; (b) ALYREF expression differences among different N stages; (c) ALYREF expression differences between M0 and M1 groups; (d) ALYREF expression differences among different clinical stages; (e) ALYREF expression differences among different grades; (f)ALYREF expression differences between female and male groups. *p < 0.05; **p < 0.01; ***p < 0.001; ****p < 0.0001.

## Data Availability

The results showed here are in whole or part based upon data generated by the TCGA Research Network: https://www.cancer.gov/tcga.
